# *Mycobacterium fortuitum* Endocarditis Associated with Cardiac Surgery, Serbia

**DOI:** 10.3201/eid1903.120763

**Published:** 2013-03

**Authors:** Dragana Vuković, Vojislav Parezanović, Branislava Savić, Ivana Dakić, Suzana Laban-Nestorović, Slobodan Ilić, Ivana Ćirković, Srdjan Stepanović

**Affiliations:** Author affiliations: University of Belgrade, Belgrade, Serbia (D. Vuković, V. Parezanović, B. Savić, I. Dakić, S. Ilić, I. Ćirković, S. Stepanović);; University Children's Hospital, Belgrade (V. Parezanović, S. Laban-Nestorović, S. Ilić)

**Keywords:** *Mycobacterium fortuitum*, endocarditis, outbreak, tuberculosis and other mycobacteria, nontuberculous mycobacteria, Serbia

**To the Editor:** *Mycobacterium fortuitum* is a member of the group of rapidly growing nontuberculous mycobacteria. It is a well-known causative agent of skin and soft tissue infections, postsurgical wound infections, and other health care–associated infections (*1*). Only sporadic cases of endocarditis caused by this bacterium have been reported (*2*–*4*). We describe a cardiac surgery–related outbreak of endocarditis caused by *M. fortuitum* in 3 children.

Over a 3-week period during 2009, eight children consecutively underwent surgery for correction of ventricular septal defect (VSD) by insertion of a bovine pericardial patch at the University Children’s Hospital in Belgrade, Serbia. None of them had previous cardiac surgery. The same patch, SJM Pericardial Patch with EnCap Technology (St. Jude Medical, St. Paul, MN, USA), was used as a source for smaller, tailored patches for all patients. Sterile scissors and forceps were used to tailor a piece of the patch needed for a corresponding VSD closure. During repeated performances of this procedure and between surgeries, the patch had been continuously stored in 2% propylene oxide (PO) provided by the manufacturer. Each tailored piece of the patch had been immersed into freshly prepared sterile saline for 6 min before defect patching. The postoperative course had been uneventful for all patients, and they were discharged 7 days after the procedure. However, 3 patients were readmitted to the hospital because of prolonged fever and increasing fatigue. Patients 1, 2, and 3 (Table) had been the fourth, sixth, and eighth patients undergoing VSD repair, respectively. Diagnosis of infective endocarditis in these patients was established by transthoracic echocardiography findings and blood cultures positive for acid-fast bacteria (Table). Acid-fast bacteria also were recovered from the patch and vegetation taken during reoperation in patient 3 (Table). The isolates were identified as *M. fortuitum* by the GenoType Mycobacterium CM assay (Hain Lifescience, Nehren, Germany) (*5*). Empiric treatment with vancomycin and ceftriaxone was switched to amikacin, ciprofloxacin, and imipenem. After 6 weeks of treatment, the patients were discharged, and all were asymptomatic 12 months later.

**Table:** Characteristics of patients in an outbreak of *Mycobacterium fortuitum* endocarditis, Serbia*

Characteristic	Patient 1	Patient 2	Patient 3
Age, y/sex	12.0/F	2.0/F	0.5/M
Comorbidity	No	Down syndrome	No
Time between surgery and readmission, d	86	97	76
Position of vegetation	Septal cusp of tricuspid valve	Septal cusp of tricuspid valve and VSD patch	Septal cusp of tricuspid valve and VSD patch
Hemodynamic consequence	Moderate tricuspid valve regurgitation	Moderate tricuspid valve regurgitation	VSD patch dehiscence
No. blood cultures collected, aerobic/anaerobic	3/3	10/4	6/3
No. blood cultures positive for acid-fast bacteria, aerobic/anaerobic	2/0	5/0	2/0
Time of collection of first positive/last positive blood culture, d after readmission	8/11	1/21	4/16
Outcome	Resolved after antimicrobial drug treatment	Resolved after antimicrobial drug treatment	Resolved after antimicrobial drug treatment and reoperation

*VSD, ventricular septal defect.

The cultural characteristics and susceptibility patterns of all the isolates obtained were indistinguishable. To explore their possible clonal relatedness, we genotyped 3 *M. fortuitum* strains isolated from blood cultures (1 isolate per patient) and 2 *M. fortuitum* isolates recovered from samples taken during reoperation in 1 of the patients. The enterobacterial repetitive intergenic consensus PCR was used (*6*), and all isolates produced identical patterns.

Nosocomially acquired *M. fortuitum* endocarditis has been reported but only sporadically in adults, and these cases usually were fatal (*3*,*4*,*7*). In contrast, we describe 3 related cases of *M. fortuitum* endocarditis in children who recovered. The relatedness of the cases is strongly supported by the following. First, epidemiologic links are obvious because the 3 patients underwent surgery in the same operating room, and the same patch was used in all of them. Second, *M. fortuitum* strains isolated from the 3 patients were phenotypically and genotypically identical.

Repeated use of the same patch in multiple surgeries strongly suggests the contaminated patch was the source of *M. fortuitum* infection in the 3 patients. This possibility could not be corroborated by bacteriologic examination of the patch because the remaining unusable fragments had been discarded after the surgeries (i.e., ≈3 months before the outbreak became evident). Although contamination of the patch during manufacture is possible (*8*), it seems more reasonable to assume that the contamination occurred intraoperatively. The common factor in nosocomially acquired *M. fortuitum* infections is presumed to be exposure to a liquid contaminated with this organism (*1*,*9*). The patch was not exposed to solutions other than the PO in which it had been stored and the sterile saline used during the rinsing procedure. Because only a piece of the patch tailored for a particular patient was exposed to a saline freshly prepared for each surgery, contamination of the PO by *M. fortuitum* presumably led to contamination of the patch. Liquid PO is used as a chemical sterilant for bioprostheses intended for single use. However, multiple use of the same patch implied repeated exposure of the PO solution to the environment and prolonged storage at 4°C between surgeries. Because PO effectiveness is markedly reduced at temperatures <16°C (*10*), the specific circumstances could have compromised the sterilizing capacity of the PO solution and enabled contamination by ubiquitous *M. fortuitum*.

We are well aware that the patch was intended for single use only and that application of the same patch in multiple patients is not a practice in industrialized countries. However, it is a practice in some resource-limited countries. The outbreak of *M. fortuitum* endocarditis we describe is a clear warning that such practice is associated with high risk and thus should be discontinued.
